# Combination of Genomic and Transcriptomic Approaches Highlights Vascular and Circadian Clock Components in Multiple Sclerosis

**DOI:** 10.3390/ijms23010310

**Published:** 2021-12-28

**Authors:** Chiara Scapoli, Nicole Ziliotto, Barbara Lunghi, Erica Menegatti, Fabrizio Salvi, Paolo Zamboni, Marcello Baroni, Francesco Mascoli, Francesco Bernardi, Giovanna Marchetti

**Affiliations:** 1 Department of Life Science and Biotechnology, University of Ferrara, 44121 Ferrara, Italy; chiara.scapoli@unife.it (C.S.); barbara.lunghi@unife.it (B.L.); marcello.baroni@unife.it (M.B.); 2 Department of Biomedical Sciences, Humanitas University, Pieve Emanuele, 20072 Milan, Italy; nicole.ziliotto@humanitasresearch.it; 3 Department of Translational Medicine and for Romagna, University of Ferrara, 44121 Ferrara, Italy; erica.menegatti@unife.it (E.M.); zambo@unife.it (P.Z.); 4 Center for Immunological and Rare Neurological Diseases, IRCCS of Neurological Sciences, Bellaria Hospital, 40139 Bologna, Italy; fabrizio.salvi@gmail.com; 5 Unit of Vascular and Endovascular Surgery, S. Anna University-Hospital, 44124 Ferrara, Italy; francesco.mascoli@unife.it; 6 Department of Neuroscience and Rehabilitation, University of Ferrara, 44121 Ferrara, Italy; giovanna.marchetti@unife.it

**Keywords:** multiple sclerosis, vascular components, GWAS, transcriptomics, WES, rare variants, circadian entrainment, circadian rhythm

## Abstract

Aiming at exploring vascular components in multiple sclerosis (MS) with brain outflow disturbance, we combined transcriptome analysis in MS internal jugular vein (IJV) wall with WES in MS families with vertical transmission of disease. Main results were the differential expression in IJV wall of 16 MS-GWAS genes and of seven genes (*GRIN2A*, *GRIN2B*, *IL20RB*, *IL26*, *PER3*, *PITX2*, and *PPARGC1A*) not previously indicated by GWAS but encoding for proteins functionally interacting with MS candidate gene products. Strikingly, 22/23 genes have been previously associated with vascular or neuronal traits/diseases, nine encoded for transcriptional factors/regulators and six (*CAMK2G*, *GRIN2A*, *GRIN2B*, *N1RD1*, *PER3*, *PPARGC1A*) for circadian entrainment/rhythm components. Among the WES low-frequency (MAF ≤ 0.04) SNPs (*n* = 7) filtered in the 16 genes, the *NR1D1* rs17616365 showed significantly different MAF in the Network for Italian Genomes affected cohort than in the 1000 Genome Project Tuscany samples. This pattern was also detected in five nonintronic variants (*GRIN2B* rs1805482, *PER3* rs2640909, *PPARGC1A* rs2970847, rs8192678, and rs3755863) in genes coding for functional partners. Overall, the study proposes specific markers and low-frequency variants that might help (i) to understand perturbed biological processes in vascular tissues contributing to MS disease, and (ii) to characterize MS susceptibility genes for functional association with disease-pathways.

## 1. Introduction

Several studies have provided evidence for vascular components participating in the complex pathogenetic network in multiple sclerosis (MS). Blood–brain barrier disruption and vascular changes, leading to cerebral hypoperfusion and tissue hypoxia, interact in a vicious cycle favoring the altered immune trafficking and the inflammatory events [1,2,3]. Most of the MS lesions have a perivascular development around small cerebral veins, a feature called “central vein sign” [4,5], which spreads across all MS clinical phenotypes. The elevation in venous pressure [6] might be a vascular feature related to the underlying pathophysiology of MS and a component of the compliance changes found in MS [7].

The cerebrovascular disease and the neurodegeneration process appear to be reciprocally linked [8,9,10], supporting alterations in neurovascular coupling mechanisms. Moreover, the occurrence of cardiovascular comorbidities [11,12,13,14] and the overlapping between genetic associations for MS and cardiovascular disease [15,16] suggest common pathological mechanisms affecting vasculature [12].

Dysfunction in the circadian clock system might contribute to neurovascular pathological conditions by affecting protein homeostasis, immune, and inflammatory functions [17,18,19,20,21,22]. Indeed, recent evidence suggests the role of molecular clocks in regulating blood–brain barrier [23], in dictating the response of immune pathways, and in modulating innate and adaptive immune crosstalk in autoimmune diseases [24]. Data support the relationships between the circadian rhythm and function of the vascular system [25], association of circadian disruption with immune activation–neurodegeneration [26], and increased incidence of MS [24].

Among hundreds of MS susceptibility genes from genome-wide association studies (GWAS) contributing to the polygenic pathways network of MS [27], many are still to be characterized in terms of functional association with MS onset/progression pathways, particularly with respect to the vascular compartment.

This study was aimed at exploring vascular components in MS by combining (i) transcriptome analysis of the internal jugular vein (IJV) walls from MS patients suffering from disturbed brain outflow [28] and (ii) whole exome sequencing (WES) in MS families [29] characterized by disturbed brain outflow and vertical transmission of MS.

## 2. Results

### 2.1. The Transcriptomic Approach: Expression Profile of MS-GWAS Genes in Vascular Tissues

The reference list of MS genes in the GWAS catalogue (*n* = 543) and the transcripts differentially expressed between MS and control IJV walls (*n* = 923) included eighteen shared records (2 ncRNA and 16 coding genes) (Table 1). Regarding the 16 coding genes (Table 2), the GO enrichment analysis by STRING (v11.0) revealed significantly (FDR ≤ 0.05) enriched terms related to immune system/response, driven by transcripts for CD86, a receptor of the immune/inflammatory response that showed the highest increase in fold change in the MS jugular wall. Transcription (biological processes) and signal transduction (reactome pathways) were also included among the significantly enriched terms. 

**Figure 1:**
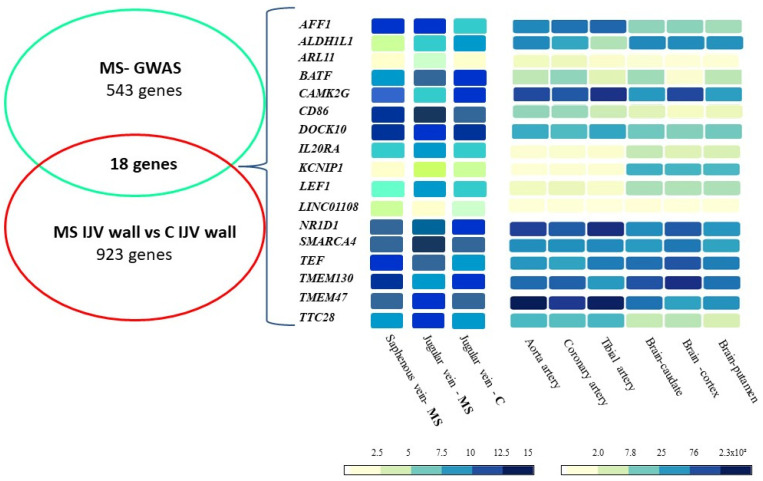
Schematic representation of the mRNA expression levels of MS-GWAS genes in vascular and brain tissues. Data from venous tissues were obtained by transcriptome analysis of IJV and SV from MS patients and from IJV of controls. Data from arteries and brain tissues were retrieved from GTEx portal. The expression levels are reported as log_2_ expression values (Table 1) for IJV and saphenous vein, or as transcript per million (TPM) for arteries and brain tissues, according to the color scales reported below the figure. For the ncRNA LOC00130476, no expression data in vascular or brain tissues are reported in GTEx portal.

In order to explore the expression at the mRNA level in other vascular tissues of the 18 selected genes, data from transcriptome analysis of saphenous vein (SV) wall, previously obtained by microarray in vascular specimens from MS patients [30], and data extracted from GTEx portal, only available for arterial vessels, were considered. In addition, the GTEx portal analysis was extended to brain, in particular the caudate, cortex, and putamen regions, of main interest for MS. The relative expression levels concerning two veins, IJV (MS patients and controls) and SV (MS patients), three arteries (aorta, coronary, and tibial), and the selected brain regions are compared in Figure 1. *CAMKG2*, *N1RD1*, *SMARCA4*, *TEF*, *TMEM130*, and *TMEM47* were characterized by high expression levels in veins, arteries, and brain regions. *BATF*, *CD86*, *IL20RA*, and *LEF1* were highly expressed in the veins and modestly in the arteries and brain regions. Differently, *ARL11*, *KCNIP1*, and *LINC01108* were expressed at very low levels in both veins and arteries, and *ARL11* and *LINC01108* also displayed low levels in brain regions. 

**Figure 2:**
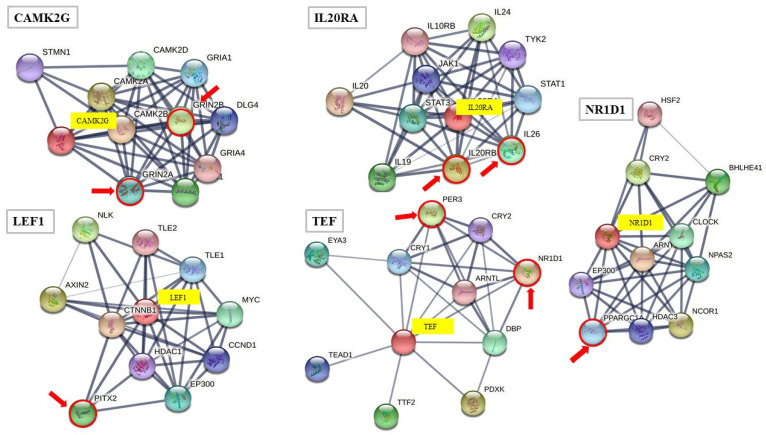
Functional partners of CAMK2G, IL20RA, LEF1, NR1D1, and TEF predicted by STRING database (v11.0). In the STRING setting, the protein nodes are reported with the corresponding gene symbol. The top ten interactors of CAMK2G, IL20RA, LEF1, NR1D1, and TEF are reported as colored nodes filled with some known or predicted 3D structure. The edges (solid lines) indicate both functional and physical protein associations. The line thickness indicates the strength of data support that ranged from 0.69 to 0.76 (high, TEF), and from 0.92 to 0.99 (highest, CAMK2G, IL20RA, LEF1, NR1D1). The genes differentially expressed between MS and C IJV walls are marked by red arrows and circles.

Functional associations of the proteins encoded by the 16 genes were explored in the STRING database, and the top 10 functional partners were considered for each gene. Five proteins (CAMKG2, IL20RA, LEF1, N1RD1, and TEF, Figure 2) were found to be functional partners (scores 0.69–0.99) of seven proteins (GRIN2A, GRIN2B, IL20RB, IL26, PITX2, PPARGC1A, and PER3) encoded by genes differentially expressed in the IJV transcriptome [28]. Among functional partners, transcriptional regulators were well represented (PITX2, PPARGC1A, PER3). Potential interactions between NR1D1 and TEF and between LEF1 and SMARCA4 (not shown in Figure 2) were also reported in the STRING database.

The DAVID (v6.8) analysis of the 12 genes, differentially expressed in the IJV transcriptome of MS patients, revealed that the terms “circadian entrainment” (KEGG pathway) and “circadian regulation of gene expression” (biological process) were significantly enriched (Benjamini-corrected *p*-value 0.011 and 0.016, respectively).

### 2.2. The Genomic–Transcriptomic Approach: WES in MS Families—Expression Profile of MS-GWAS Genes

**Figure 3:**
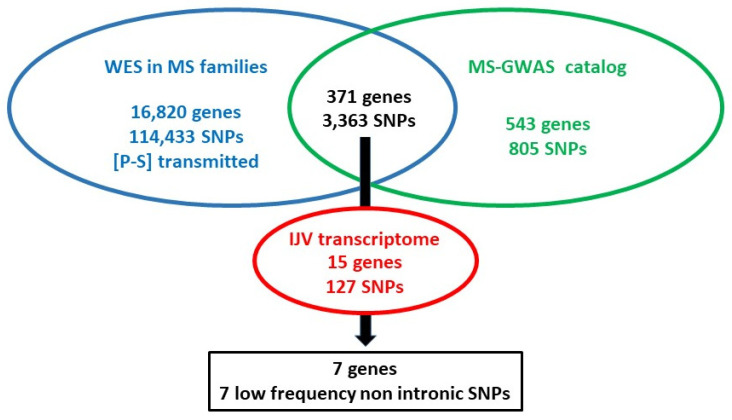
Schematic representation of the filtering approaches to prioritize SNPs. Variants with vertical transmission of disease within the MS families (WES output, blue) were selected and then filtered using both MS-GWAS (green) and transcriptomic (red) datasets.

Data from the targeted WES-based pilot analysis in MS families were examined to identify variants showing vertical transmission of disease within families. A total of 114,433 single-nucleotide polymorphisms (SNPs) were observed in 16,820 genes (Figure 3). To prioritize SNPs combining genomic and transcriptomic approaches, data from WES output in families were filtered with both MS-GWAS and transcriptomic sets as schematized in Figure 3, leading to the selection of 127 variants in 15 genes (Figure 3) among the 18 selected by transcriptomic GWAS approach (Table 1). We focused on low-frequency (MAF ≤ 0.04) and nonintronic variants (Figure 3, Table 3 and Table S1, MS-GWAS genes), and extended the investigation of disease association by using data from i) the Network for Italian Genomes “NIG-IT” that includes individuals affected by different diseases, among which includes MS, and ii) controls from gnomAD 3.1.1 (nfe) and from Tuscany in the 1000 Genome Project. Among the seven prioritized SNPs, the *DOCK1* rs113265459, the *NR1D1* rs17616365, and *TMEM130* rs199556348 showed significantly higher MAF in the NIG-IT affected cohort than in the 1000G TSI samples (Table 3). MAF of *NR1D1* and *TMEM130* variants in the NIG-IT Italian sample was significantly different from that in the gnomAD 3.1.1. 

The expression quantitative trait loci (eQTL) analysis (GTEx portal), focused on vascular and brain tissues, supported the functional impact of eight SNPs among the MS-GWAS low-frequency nonintronic variants (*n* = 2) and among nonintronic variants (*n* = 6) in functional partner genes (Table S1). 

The *NR1D1* rs17616365 (Table 3 and Table S1, MS-GWAS genes) affects the mRNAs of CASC3, a protein of the exon–junction complex, and of WIPF2, a protein involved in the organization of cytoskeleton. Further, the 5′UTR *NR1D1* rs17616365 has been associated with sex hormone-binding globulin levels. The *CD86* rs11575853 (Table S1) affects the expression of mRNA encoding NPHP5, a nephrocystin protein that interacts with calmodulin and the retinitis pigmentosa GTPase regulator protein. The stop codon gains of *ARL11* rs34301344 and the amino acid substitution (Val145Leu) of *LEF1* rs141850161 could also mark functional variants (Table S1). 

The detection of variants by WES, extended to the genes (*GRIN2A*, *GRIN2B*, *IL20RB*, *IL26*, *PITX2*, *PPARGC1A*, and *PER3*) encoding the predicted functional partners (Figure 2) revealed 57 SNPs, 39 intronic and 18 nonintronic. The analysis of the nonintronic SNPs (Table 3, functional partners) highlighted five variants (*GRIN2B* rs1805482, *PER3* rs2640909, *PPARGC1A* rs2970847, rs8192678, and rs3755863) differing significantly in frequency (*p* < 0.0008) between the NIG-IT cohort and the 1000G TSI. For two polymorphisms, *PER3* rs2640908 and *GRIN2B* rs7301328, a borderline significance was observed. When compared with gnomAD controls, the *PER3* rs2640909 and *PPARGC1A* rs3755863 showed a borderline or nominal significant *p*-value, respectively. 

The eQTL analysis for the nonintronic SNPs in functional partners (Table 3 and Table S1, functional partners) showed that three *PER3* variants (the synonymous rs228669 and two missense rs228696 and rs228697) affect the expression of the PER3 mRNA. Moreover, four *PER3* variants (two synonymous rs2640908, rs2859387 and two missense rs2640909, rs228696) affect the expression of VAMP3 mRNA, a protein of the complex involved in docking/fusion of synaptic vesicles. The *PER3* rs2859387 also affects the splicing (sQTL) of VAMP3 mRNA and the *PER3* rs2640908 the mRNA levels of UTS2, a vasoconstrictor peptide (Table 3 and Table S1, functional partners).

### 2.3. Associations with Vascular/Neuronal Traits/Diseases

**Figure 4:**
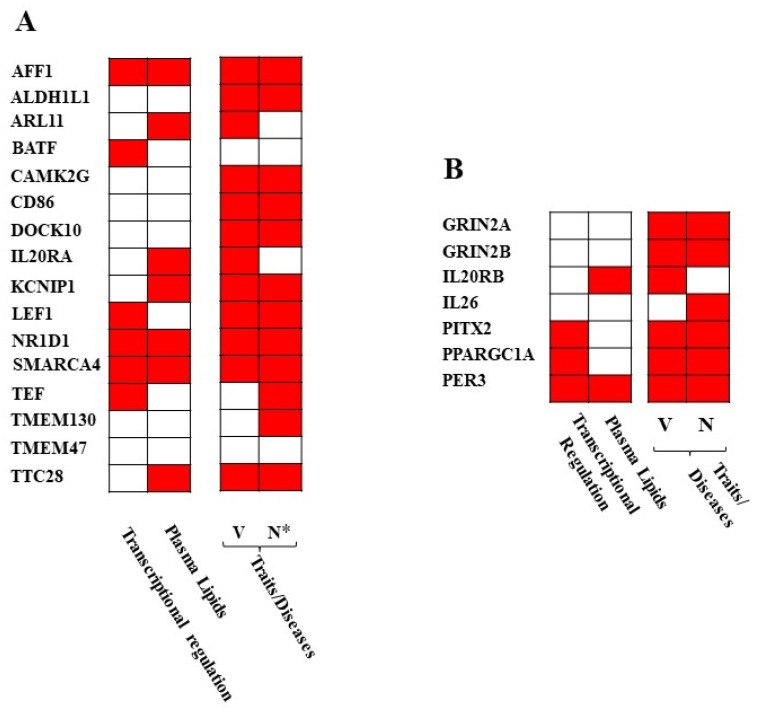
Association of GWAS (**A**) and interactor (**B**) genes and their encoded mRNA/proteins with transcription, and with vascular (V)/neuronal (N) traits or diseases according to literature and databases. N*, neuronal diseases additional to MS. Associations of ncRNAs (LINC01108 and LOC00130476) with diseases were not found in literature and databases.

To provide insights into association of selected genes/proteins with diseases, particularly with vascular and neuronal aspects, the associations of the 16 GWAS coding genes and of the seven functional partners were investigated by bioinformatics (DAVID v6.8). Although the terms “pharmacogenomics”, “immune”, and “hematological” were listed in DAVID, none reached significance after Benjamini correction. In literature mining, for five out of the 16 genes (*BATF*, *CD86*, *IL20RA*, *KCNIP1*, *LEF1*), association with MS was detected also in studies conducted at RNA or protein levels (Table 4). Both vascular and neuronal associations, additional to MS, were detected for 11 out of 16 genes, and eight were also associated with plasma lipid levels (Figure 4A). Interestingly, several of these genes were immune-related and one of them (*LEF1*) has been previously related to the blood–brain barrier (Table 4).

Virtually all the functional partners showed associations with vascular traits/diseases and with neuronal traits/diseases, including MS (Figure 4B, Table S2). To note, *GRIN2A*, *GRIN2B*, *IL20RB*, *IL26*, *PITX2*, *PPARGC1A*, and *PER3* are not present in the MS-GWAS catalog.

## 3. Discussion

We propose candidate vascular components in MS by combining the transcriptional pattern in the wall of IJV, the main cerebral venous outflow pathway [67], with MS-GWAS and analysis of vertically transmitted disease variants within three multi-incidence MS families with disturbed brain outflow. Within GWAS genes with differential expression, we focused on low-frequency variants, which are not individually detectable at genome-wide thresholds but are potentially endowed with functional impact [15,68]. Noticeably, the analysis, extended to the non-MS-GWAS functional partners of transcriptionally dysregulated genes, highlighted numerous associations with vascular or neuronal traits/diseases, including MS. 

### 3.1. Transcription Factor Expression—Vascular Function Alteration in MS-Cardiovascular Risk Traits 

Genomic and transcriptomic approaches pointed out a noticeable proportion of genes (9/23), MS-GWAS, and functional interactors encoding for transcription factors or regulators, which could bridge alteration of vascular function with MS disease. Moreover, finding virtually all selected genes (22/23) associated with vascular or neuronal traits/diseases strongly supports the presence of pleiotropic loci between MS and cardiovascular comorbidities.

LEF1 and SMARCA4 belong to the WNT/beta-catenin pathway, upregulated in demyelinating events characterizing neurodegeneration [69] and modulating the immune response in MS. LEF1, a brain endothelium-specific transcription factor, has been associated [54] with vascular remodeling and post-stroke angiogenesis [55], and SMARCA4 [70] with cardio–cerebral–peripheral vascular diseases in GWAS [61]. 

*SMARCA4*, *AFF1*, and *NR1D1*/Rev-Erbα provide a link between immunity-lipid metabolism–vascular function. *SMARCA4* and *AFF1* contain several SNPs associated with triglyceride and cholesterol plasma levels [61,71], suggesting the genetic overlap between blood lipids and immune-mediated diseases [72]. *NR1D1*/Rev-Erbα may exert atheroprotective action through inhibition of (i) the human *APOC3* promoter gene activity [58] and (ii) PAI1 expression [59], a major negative regulator of fibrinolysis. 

*CAMKG2*/CaMKIIγ, a component of the Ca^2+^-dependent signaling pathways that influences proliferation of vascular smooth muscle cells and vascular remodeling [37], is shared between two major neurological diseases: ischemic stroke and MS [73]. 

CD86, driving the atherogenic process [74] in the adaptive immune system, and BATF relate MS disease with cardiovascular disease risk factors, high-density lipoprotein cholesterol (CD86), and systolic blood pressure (BATF) [15]. To note, the 5′UTR *CD86* rs11575853 was highlighted in the MS families under study and has been found associated with lower expression in several arterial tissues and brain regions of the MS-GWAS gene *IQCB1*, which encodes for the nephrocystin NPHP5. An intriguing hypothesis is that the altered expression of NPHP5, involved in amaurosis and retinopathies [75], represents a potential link between retinal diseases and neurodegeneration [76].

### 3.2. NR1D1–PPARGC1A–PER3 Expression: Vascular and Circadian Clock Components in MS

**Figure 5:**
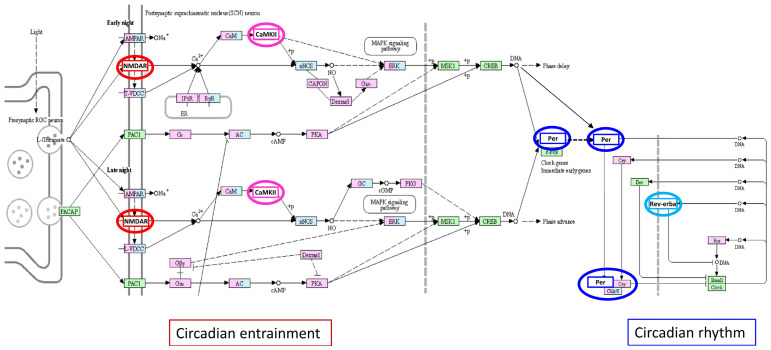
The “circadian entrainment” and “circadian rhythm” KEGG pathway members encoded by genes differentially expressed in the wall of IJV (*CAMK2G*, *GRIN2A*, *GRIN2B*, *PER3*, *NR1D1*). In the KEGG graph, the proteins encoded by these genes are marked as colored circles: the red circle indicates the glutamate ionotropic receptor NMDA that contains subunit 2A and 2B coded by *GRIN2A*, *GRIN2B*; the pink circle indicates the CaMKIIγ protein kinase coded by *CAMK2G*; the blue circle indicates the PER proteins among which the period circadian clock 3 is coded by *PER3*; the light blue circle indicates the Rev-Erbα protein coded by *NR1D1*. In accordance with KEGG color codes, pink rectangles indicate disease genes; light blue rectangles indicate drug target; pink/light blue rectangles indicate disease gene and drug target elements; green rectangles indicate specific signaling pathway elements.

Several lines of evidence support the involvement of circadian rhythm in the pathogenesis of many neurodegenerative diseases, including MS [20,22]. We highlight differential expression in the wall of IJV, which drains blood from the brain, of several circadian entrainment/rhythm components (Figure 5). These further support relationships between the complex circadian network and MS disease. Of interest, *NR1D1*, highlighted both by the MS GWAS/IJV transcriptome and GWAS/MS family transmission analyses, provides another piece of the complex neurovascular network in MS, represented by the circadian clock. Indeed, *NR1D1*/Rev-Erbα negatively regulates the expression of circadian clock proteins. Evidence has been provided for relationships between the circadian rhythm and function of the vascular system [25], and for association of circadian disruption with immune activation–neurodegeneration [26] and increased incidence of MS [24]. The *NR1D1* 5′UTR rs17616365 is associated in brain and arterial tissues with the mRNA levels of CASC3, a protein found to participate to a regulatory network in MS [77].

Noticeably, two other genes, *PPARGC1A* and *PER3*, not reported in the MS-GWAS catalog and involved in the circadian clock, both functional partners of NR1D1, were found differentially expressed in MS vs. control jugular wall and, in families with disturbed brain blood outflow, harbored SNPs with intriguing links to vascular aspect in MS. 

PPARGC1A, a transcriptional coactivator, represents a key component of the circadian oscillator that integrates the mammalian clock and energy metabolism [78]. The *PPARGC1A* rs8192678 (Gly482Ser) was very recently found to confer an increased risk for the occurrence of MS and lipid/metabolic disorders [79]. The finding of lower mRNA levels in MS than in control IJV walls is compatible with the reduced mRNA expression in peripheral blood mononuclear cells of RR-MS [80] and with reduced expression of PPARGC1A and neuronal loss in MS cortex [81].

PER3, a transcriptional repressor, has been associated with homeostatic regulation of sleep, circadian clock phenotypes [82], and immune response [83].

The *PER3* rs2640909 (Met1037Thr) and rs2640908 (synonymous), not in linkage disequilibrium, were found to predict in arterial tissues the expression levels of the adjacent *VAMP3*, involved in synaptic vesicles biology and potential target for preventing disturbed flow-dependent thrombus formation [84]. The relationship of *PER3* variations/circadian clock/MS is supported by a *PER3* VNTR polymorphism, found to impact the sleep disturbances in MS [85].

As the disruption of circadian rhythms can lead to dysregulation of cellular and molecular mechanisms of the immune cells, and therefore contribute to aberrant immune response [17,18], finding signals in genes belonging to this complex circadian network in MS is of great interest.

A limitation of this study is the low number of investigated patients and families, who, on the other hand, were selected for disturbed brain outflow. Based on these features, the selected genes and markers could be more tightly associated with a subgroup of MS patients.

## 4. Conclusions

Based on transcriptome and genomic analysis in MS, our study highlights the differential expression in venous tissue, particularly in the wall of the IJV, which drains blood from the brain, of 16 MS-GWAS genes, and of seven genes not previously indicated by GWAS, but functionally interacting with MS candidates. Virtually all genes have been previously associated with vascular or neuronal traits/diseases additional to MS and nine encoded for transcriptional factors/regulators. Eight genes were associated with plasma lipid levels supporting the link between lipids and immune-mediated diseases. 

Differential expression and frequency of low-frequency variants were observed for six genes involved in circadian entrainment/rhythm. 

Future studies should focus on the functional characterization of selected markers and SNPs (i) “in vitro”, by recombinant expression of SNPs which predict alteration/modulation of the gene/protein expression; (ii) in plasma/WBC of MS patients to evaluate protein/mRNA [86] with predicted alteration/modulation (i.e, plasma lipids), and (iii) in patients undergoing exercise and rehabilitation strategies [87], grouped for specific alleles of the circadian entrainment/rhythm genes.

## 5. Materials and Methods

The list of genes associated with MS was created by the NHGRI-EBI GWAS catalogue [88]. SNPs listed under “multiple sclerosis” and with *p*-value ≤ 5 × 10^−6^ were selected. Based on these selected common variants, the reference gene list for the present study included 805 SNPs attributable to 543 genes. 

Microarray-based transcriptome analysis of IJV walls has been already described [28]. The list of differentially expressed genes between MS- and control-IJV walls (fold change ≥2, *p* ≤ 0.05, multiple testing correction), included 923 genes [28]. 

Expression data of selected genes in arterial vessels and brain regions were extracted from Genotype-Tissue Expression (GTEx) portal [89].

For selected genes, gene ontology enrichment analysis (FDR ≤ 0.05) for biological processes, molecular functions, and reactome pathways was conducted with STRING [90]. Functional partners (protein–protein interaction, PPI) were explored by STRING database (v11.0, https://version-11-0b.string-db.org/, accessed on 1 July 2021). In the STRING setting, the protein nodes are reported with the corresponding gene symbol and as colored nodes filled with some known or predicted 3D structure. The network edges were reported with the “confidence” mode (solid lines). The line thickness indicates the degree of confidence prediction of the interaction (low 0.150; medium 0.400; high 0.700; highest 0.900). Only the top ten (high to highest) interactors were included in the analysis. The Database for Annotation, Visualization and Integrated Discovery (DAVID, https://david.ncifcrf.gov/home.jsp, accessed on 10 November 2021) v6.8 [91] was used to perform functional enrichment analyses of the differentially expressed functional partner and related genes. Significantly enriched terms were defined as having Benjamini-corrected *p*-value < 0.05. 

Pathways of molecular interactions and relations of selected genes were obtained from Kyoto Encyclopedia of Genes and Genomes (KEGGS) portal [92].

A WES was performed on 11 individuals, seven diagnosed with MS and four unaffected, from three independent families. Sequencing was performed by BGI (Shenzhen, China) using nanoarray-based short-read sequencing-by-ligation technology (cPALTM) as previously described [29].

To identify new exonic and/or potentially functional variants we firstly analyzed data from the targeted WES-based pilot analysis in MS families to identify all variants showing vertical transmission of disease within families. Then, we filtered these SNPs with both MS-GWAS and transcriptomic sets. To prioritize the selected variants, we investigated these candidate SNPs in publicly available datasets of human DNA sequence variation (1000 Genomes Project [93]; Genome Aggregation Database gnomAD [94]). 

Based on the higher genetic variability in the Italian population as compared with other European populations [95,96], leading to recommending the use of population-specific databases for a more accurate assessment of variant pathogenicity [97], we compared populations with MAFs obtained from (i) the Network for Italian Genomes “NIG-IT” which include whole-exome sequencing for 1098 unrelated Italian individuals affected by different diseases, MS included; (ii) Controls from 1000 Genome Project which contains allelic frequencies for a sample of Tuscany, Italy (1000G TSI), an optimal reference population for individuals under study. The low prevalence (188 per 100,000 individuals) of MS in Tuscany [98] makes improbable the presence of individuals with MS in the Tuscany control sample. Although the 1000G TSI cohort is the most closely related to our sample, it is too small to produce accurate frequencies for low-frequency variants. Thus, we used the non-Finnish European (nfe) populations from gnomAD 3.1.1 as a further control population. 

To test the difference in MAFs between reference and study populations, a two-proportion z-test with a 0.05 two-sided significance level was applied. A threshold of *p* < 0.002, assuming the Bonferroni correction for multiple testing, was used for significance.

## Figures and Tables

**Table 1:** MS-GWAS genes differentially expressed between MS and control IJV walls.

Gene Symbol	Gene Name	Regulation	MS-IJV	C-IJV	FC	*p* *
*AFF1*	AF4/FMR2 family member 1	up	9.95 ± 0.30	8.84 ± 0.29	2.17	0.005
*ALDH1L1*	Aldehyde dehydrogenase 1 family member L1	down	6.03 ± 0.22	7.23 ± 0.36	2.29	0.004
*ARL11*	ADP ribosylation factor-like GTPase 11	up	3.11 ± 0.42	2.07 ± 0.32	2.06	0.010
*BATF*	Basic leucine zipper ATF-like transcription factor	up	10.52 ± 1.39	8.94 ± 0.46	2.99	0.049
*CAMK2G*	Calcium/calmodulin dependent protein kinase II gamma	down	6.80 ± 0.57	8.01 ± 0.48	2.31	0.019
*CD86*	CD86 molecule	up	14.38 ± 0.89	11.30 ± 0.38	8.48	0.002
*DOCK10*	Dedicator of cytokinesis 10	down	8.71 ± 0.98	10.01 ± 0.44	2.47	0.038
*IL20RA*	Interleukin 20 receptor subunit alpha	up	7.57 ± 1.23	5.97 ± 0.39	3.00	0.034
*KCNIP1*	Potassium voltage-gated channel interacting protein 1	up	3.84 ± 0.66	2.65 ± 0.70	2.28	0.042
*LEF1*	Lymphoid enhancer binding factor 1	up	7.64 ± 0.44	6.29 ± 0.96	2.55	0.042
*LINC01108*	ncRNA	down	2.09 ± 0.54	3.17 ± 0.60	2.12	0.034
*LOC100130476*	ncRNA	up	3.33 ± 0.15	2.16 ± 0.35	2.24	0.047
*NR1D1*	Nuclear receptor subfamily 1 group D member 1	up	8.31 ± 0.97	6.45 ± 1.16	3.63	0.042
*SMARCA4*	SWI/SNF related, matrix associated, actin dependent regulator of chromatin, A4	up	14.07 ± 0.86	12.12 ± 0.33	3.86	0.007
*TEF*	Thyrotroph embryonic factor	up	9.02 ± 0.47	7.76 ± 0.91	2.40	0.045
*TMEM130*	Transmembrane protein 130	down	6.73 ± 0.31	7.94 ± 0.60	2.32	0.016
*TMEM47*	Transmembrane protein 47	down	9.60 ± 0.82	10.70 ± 0.54	2.21	0.043
*TTC28*	Tetratricopeptide repeat domain 28	up	8.72 ± 0.54	7.52 ± 0.33	2.30	0.011

MS-IJV and C-IJV columns, mean ± SD of the log-transformed (log_2_) expression values. The fold change (FC) is presented as an absolute value. * by moderate t-test, followed by the application of Benjamini–Hochberg multiple testing correction.

**Table 2:** Names and function of proteins encoded by MS-GWAS genes differentially expressed between MS and control IJV walls.

Gene Symbol	Protein Name (Short Name)	Protein Function
*AFF1*	AF4/FMR2 family member 1 (AF4)	Transcription factor, elongation complex of RNA pol II (https://www.ncbi.nlm.nih.gov/gene/4299)
*ALDH1L1*	Cytosolic 10-formyltetrahydrofolate dehydrogenase (ALDH1L1, FDH)	Enzyme of folate metabolism (https://www.ncbi.nlm.nih.gov/gene/10840)
*ARL11*	ADP ribosylation factor-like protein 11 (ARLTS1)	GTP-binding protein (https://www.ncbi.nlm.nih.gov/gene/115761)
*BATF*	Basic leucine zipper transcriptional factor ATF-like (B-ATF)	Transcription factor (https://www.ncbi.nlm.nih.gov/gene/10538)
*CAMK2G*	Calcium/calmodulin-dependent protein kinase type II subunit gamma (CAMK-II gamma)	Enzyme, serine/threonine protein kinase, calcium signaling (https://www.ncbi.nlm.nih.gov/gene/818)
*CD86*	T-lymphocyte activation antigen CD86 (CD86)	Receptor, type 1 membrane protein https://www.ncbi.nlm.nih.gov/gene/942
*DOCK10*	Dedicator of cytokinesis 10 (DOC-10)	Guanosine nucleotide exchange factor (https://www.ncbi.nlm.nih.gov/gene/55619)
*IL20RA*	Interleukin-20 receptor subunit alpha (IL-20R-alpha)	Receptor of IL20, membrane protein (https://www.ncbi.nlm.nih.gov/gene/53832)
*KCNIP1*	Kv channel-interacting protein 1(KChIP1)	Neuronal membrane excitability (https://www.ncbi.nlm.nih.gov/gene/30820)
*LEF1*	Lymphoid enhancer-binding factor 1 (LEF1)	Transcription factor, WNT-signaling (https://www.ncbi.nlm.nih.gov/gene/51176)
*NR1D1*	Nuclear receptor subfamily 1 group D member 1 (EAR-1) Rev-erbA-alpha	Heme-dependent transcriptional repressor (https://www.ncbi.nlm.nih.gov/gene/9572)
*SMARCA4*	Transcription activator BRG1 (BAF190A)	Enzyme, helicase and ATPase activities, regulator of transcription (https://www.ncbi.nlm.nih.gov/gene/6597)
*TEF*	Thyrotroph embryonic factor	Transcription factor that binds to and transactivates the TSHB promoter (https://www.ncbi.nlm.nih.gov/gene/7008)
*TMEM130*	Transmembrane protein 130	Membrane-bound signaling protein (https://www.ncbi.nlm.nih.gov/gene/222865)
*TMEM47*	Transmembrane protein 47	Adherens junction, cell–cell adhesion (https://www.ncbi.nlm.nih.gov/gene/83604)
*TTC28*	Tetratricopeptide repeat protein 28	Scaffold-adaptor protein (https://www.ncbi.nlm.nih.gov/gene/23331)

The gene symbols are reported according to NCBI Gene database (https://www.ncbi.nlm.nih.gov; accessed on 20 December 2021). The recommended protein names and the reported short names (parenthesis) are in accordance with the UniProt Knowledgebase. The molecular function of the proteins is reported according to the Report of the corresponding gene in NCBI Gene database. Noticeably, one third of transcripts upregulated in MS jugular walls encoded for transcription factors (AFF1, BATF, LEF1, NR1D1, and TEF) or for a transcription regulator, SMARCA4, an ATP-dependent chromatin remodeling protein (Table 2).

**Table 3:** Nonintronic variants with significant differences in allele frequency between affected and control Italian cohorts.

**Gene**	**Chr: Position GRCh38** **dbSNP**	**Ref. Allele**	**mRNA** **Molecular Consequences**	**mRNA Level Affected by SNP eQTL**	**CADD (°)** **(Ensembl)**	**NIG-IT** **MAF**	**1000G** **TSI** **MAF**	**gnomAD 3.1.1 (nfe)** **MAF**	***p*-Value ($)** **NIG-IT vs. 1000G TSI**	***p*-Value ($)** **NIG-IT vs. gnomAD 3.1.1**
MS-GWAS genes										
*DOCK10*	chr2:224795011:G:A/C rs113265459	A	caC/caT p.His1674His	ns	A:5.937	0.0224	0	0.0165	**6.87 × 10^−7^**	0.1296
*NR1D1*	chr17:40100148:G:A rs17616365	A	c.-54C > T 5′-UTR	CASC3, WIPF2	A:16.11	0.0119	0.00485	0.0283	**0.00083**	**0.0011**
*TMEM130*	chr7:98863351:C:T rs199556348	T	gcG/gcA p.Ala45Ala	ns	T:1.532	0.00046	0.00002	0.00003	**0.0013**	**0.0094**
Functional partners										
*GRIN2B*	chr12:13611840:G:A rs1805482	A	agC/agT p.Ser555Ser	ns	A:0.955	0.3215	0.3786	0.3377	**0.00011**	0.2569
*GRIN2B*	chr12:13865843:G:C/T rs7301328	C	ccC/ccG p.Pro122Pro	ns	C:7.840	0.3646	0.4126	0.3892	**0.00131**	0.0958
*PER3*	chr1:7829881:C:T rs2640908	T	acC/acT (+) p.Thr978Thr	VAMP3, UTS2	T:0.757	0.2044	0.2379	0.1918	**0.0093**	0.2884
*PER3*	chr1:7830057:T:C rs2640909	C	aTg/aCg (+) p.Met1037Thr	VAMP3	C:0.161	0.2500	0.3204	0.2872	**7.27 × 10^−7^**	**0.0067**
*PPARGC1A*	chr4:23814301:T:C/A rs2970847	C	acA/acG p.Thr394Thr	ns	C:1.605	0.1763	0.1408	0.1923	**0.00077**	0.1791
*PPARGC1A*	chr4:23814039:C:T rs8192678	T	Ggt/Agt p.Gly482Ser	ns	T:16.24	0.3572	0.4272	0.3450	**3.33 × 10^−6^**	0.3964
*PPARGC1A*	chr4:23813899:C:T/A/G rs3755863	T	acG/acA p.Thr528Thr	ns	T:9.102	0.4380	0.5097	0.4054	**2.42 × 10^−6^**	0.0287

Genomic coordinates from NCBI Build GRCh38.p13 (hg38) and dbSNP refSNP (rs) identifiers from build 155 are provided. mRNA levels significantly affected by SNPs in vascular and/or brain tissues (reported in GTEx portal as expression quantitative traits, eQTL) are provided. ns, no significant eQTL was found. CASC3, exon junction complex subunit GJD3, involved into spliceosomes at the exon–exon junction; WIPF2, WAS/WASL interacting protein family member 2, involved in the WASP-mediated organization of the actin cytoskeleton. VAMP3, a protein involved in docking/fusion of synaptic vesicles; UTS2, urotensin 2. Minor allele frequency (MAF) from WES of affected subjects collected by the Network for Italian Genomes (NIG-IT); controls from 1000 Genome Tuscany (TSI) and gnomAD 3.1.1 non-Finnish European (nfe) databases are given. ($) Bonferroni’s multiple test correction was set to *p* < 0.002: in red, SNPs significant after Bonferroni correction; in blue, SNPs at borderline significance after Bonferroni correction; in black, nominally significant variants. (°) Estimated effect on protein function was assessed with the Combined Annotation-Dependent Depletion (CADD) phred-scale scores v1.4 (for convenience, scores above 30 are regarded as “likely deleterious” and scores below as “likely benign”).

**Table 4:** MS- GWAS genes differentially expressed between MS and control IJV walls: association with neuro/vascular processes, traits, and diseases.

Gene	Protein Function	Gene/SNPs-Associated Processes/Traits/Diseases	
		Vascular	Neuronal
*AFF1*	Transcription factor; elongation complex RNA pol II	TG, HDL-LDL cholesterol levels, CAD [31]	Cognitive decline in AD, glioblastoma [32]
*ALDH1L1*	Enzyme; folate metabolism	Aortic stenosis, ischemic stroke [33]	Neural tube defects [34]
*ARL11*	GTP- binding protein, tumor suppressor gene	Total cholesterol levels [35]	nr
*BATF*	Transcription factor	Blood pressure [15]	MS* [36]
*CAMK2G*	Enzyme; calcium signaling	VSMC proliferation and vascular remodeling [37]	AD, intellectual developmental disorder [38]
*CD86*	Receptor, immune-inflammatory response	HDL-cholesterol, atherogenic process, stroke, vascular rejection [15,39]	MS* [40,41], autoimmune peripheral neuropathy [42]
*DOCK10*	Guanyl–nucleotide exchange factor activity	Circulating thrombin activatable fibrinolysis inhibitor [43], platelet count	Dendritic spine morphogenesis, innate immunity and neuroinflammation in EAE, autism [44,45]
*IL20RA*	Receptor of IL20, membrane protein	HDL-LDL cholesterol levels, plasma trimethyllysine # [46,47]	MS* [48], neuroinflammation in EAE [49]
*KCNIP1*	Ion transmembrane transport	TG levels; atrial fibrillation, hypertension, heart rate in heart failure [50,51]	MS* [52], neuronal calcium sensor, neuronal membrane excitability, ALS, schizophrenia [53]
*LEF1*	Transcription factor; WNT-signaling	Maturation of the blood–brain barrier, vascular remodeling, and angiogenesis [54,55]	MS* [56], schizophrenia, sporadic ALS [57]
*NR1D1*	Transcription factor (REV-ERBα); transcriptional repressor, circadian clock component	Lipoproteins levels, anti-inflammatory and atheroprotective action, negative regulation of PAI-1 [58,59]	Neuroinflammation -neurodegeneration, autism [26,60]
*SMARCA4*	Enzyme, helicase and ATPase activities, regulator of transcription	LDL cholesterol levels, CAD, MI, stroke, carotid atherosclerosis, PAD [31,61]	Growth retardation and neuronal degeneration, ALS [62,63]
*TEF*	Transcription factor	nr	Depressive disorders, PD [64]
*TMEM130*	Membrane-bound signaling protein	nr	Autism [65]
*TMEM47*	Adherens junction, cell–cell adhesion	nr	nr
*TTC28*	scaffold- adaptor protein	TG levels, systolic blood pressure [31]	Cerebrospinal T-tau levels [66]

MS*, association with MS detected also in RNA/protein studies; #, plasma trimethyllysine are associated with progression of atherosclerosis and myocardial infarction (MI) risk; AD, Alzheimer’s disease; ALS, amyotrophic lateral sclerosis; EAE, experimental autoimmune encephalomyelitis; PAD, peripheral artery disease; PD, Parkinson’s disease; TG, triglycerides; VSMC, vascular smooth muscle cell; nr, not reported in literature.

## Data Availability

The “NIG—Network for Italian Genomes” database is freely available at the following link http://www.nig.cineca.it/, accessed on 10 November 2021.

## Supplementary Materials

The following are available online at https://www.mdpi.com/article/10.3390/ijms23010310/s1.
