# Supplementary material for: Combination of Genomic and Transcriptomic Approaches Highlights Vascular and Circadian Clock Components in Multiple Sclerosis
Source: Int J Mol Sci. 2021 Dec 28;23(1):310. doi: 10.3390/ijms23010310 (PMC8745220; doi:10.3390/ijms23010310)
Supplement: Supplementary file 1 [file ijms-23-00310-s001.zip › Table_S1.pdf]

Table S1. Non intronic variants observed among low-frequency (MAF≤0.04) SNPs in MS-GWAS candidate genes (upper part) or among SNPs (any MAF) detected in functional partners (lower part) and showing vertical transmission of disease within families in multi-incident families.

Gene	Chr: Position in GRCh38 dbSNP	MAF allele	mRNA molecular consequences	mRNA levels affected by SNP	CADD (ensembl)	NIG-IT MAF	1000G TSI MAF	gnomAD 3.1.1 (nfe) MAF	p-value($) NIG-IT vs TSI	p-value($) NIG-IT vs gnomAD 3.1.1
**MS- GWAS genes**										
AFF1	chr4:87135773:A:G rs342464	A	c.*72A>G 3’ UTR	ns	G:0.211	0	0	0	-	-
ARL11	chr13:49630893:G:A rs34301344	A	tGg/tAg p.Trp149Ter	ns	A:40.00	0.02331	0.02913	0.01081	0.2532	**6.97E-05**
CD86(*)	chr3:122055434:A:G rs11575853	G	c.-56A>G 5’ UTR	NPHP5	G:5.951	0.03102	0.02913	0.03239	0.7106	0.7983
DOCK10	chr2: 224795011:G:A/C rs113265459	A	caC/caT p.His1674His	ns	A:5.937	0.02235	0	0.0165	**6.87E-07**	0.1296
LEF1	chr4:108089239:C:A rs141850161	A	Gtg/Ttg p.Val145Leu	ns	A:24.50	-	0	0.0008764	-	-
NR1D1	chr17: 40100148:G:A rs17616365	A	c.-54C>T 5’ UTR	CASC3, WIPF2	A:16.11	0.01190	0.00485	0.0283	**0.00083**	**0.0011**
TMEM130	chr7: 98863351:C:T rs199556348	T	gcG/gcA p.Ala45Ala	ns	T:1.532	0.00046	0.00002	0.00003	**0.0013**	**0.0094**
**Functional partners**										
GRIN2A	chr16:9822347:C:G/T rs9806806	G	cgG/cgC p.Arg695Arg	ns	G:9.893	0.24245	0.2767	0.2714	**0.0116**	**0.0318**
GRIN2A	chr16:9849809:C:T rs2229193	T	ctG/ctA p.Leu425Leu	ns	T:6.767	0.24428	0.2670	0.2862	0.0901	**0.0022**
GRIN2B	chr12:13563704:G:A/T rs1806191	A	caC/caA p.His1178His	ns	A:5.765	0.45662	0.4854	0.5000	0.05743	**0.0042**
GRIN2B	chr12:13611840:G:A rs1805482	A	agC/agT p.Ser555Ser	ns	A:0.955	0.32146	0.3786	0.3377	**0.00011**	0.2569
GRIN2B	chr12:13865843:G:C/T rs7301328	C	ccC/ccG p.Pro122Pro	ns	C:7.840	0.36459	0.4126	0.3892	**0.00131**	0.0958
GRIN2B	chr12:13866223:C:T rs12818068	T	c.-15G>A 5’ UTR	ns	T:16.74	0.07397	0.07767	0.1130	0.6480	**5.00E-05**
IL26	chr12:68225824:G:A rs11570915	A	n.-8705G>A Upstream	ns	A:12.57	0.14822	0.1650	0.1291	0.1357	0.0599
PER3	chr1:7809988:T:C rs228669	T	agT/agC p.Ser446Ser	PER3	C:1.668	0.07976	0.0874	0.0758	0.3702	0.6202
PER3	chr1:7827433:T:C/A/G rs228696	T	cTg/cCg p.Leu827Pro	PER3, VAMP3	C:6.605	0.04645	0.0631	0.0442	**0.0239**	0.7117
PER3	chr1:7827188:G:A/C rs2859387	A	ccG/ccA p.Pro753Pro	VAMP3	A:0.420	0.32240	0.3204	0.3551	0.8874	**0.0242**
PER3	chr1:7829881:C:T rs2640908	T	acC/acT p.Thr978Thr	VAMP3, UTS2	T:0.757	0.20442	0.2379	0.1918	**0.0093**	0.2900
PER3	chr1:7827519:C:G rs228697	G	Cct/Gct p.Pro864Ala	PER3	G:0.786	0.12762	0.1505	0.1020	**0.0348**	**0.0053**
PER3	chr1:7837129:A:G rs35072750	G	Act/Gct p.Thr1177Ala	ns	G:13.30	0.00137	0.004854	0.001605	0.0981	0.8463
PER3	chr1:7830057:T:C rs2640909	C	aTg/aCg p.Met1037Thr	VAMP3	C:0.161	0.2500	0.3204	0.2872	**7.27E-07**	**0.0067**
PPARGC1A	chr4:23884700:G:T/A rs2946385	T	Cga/Aga p.Arg96Arg	ns	T:0.050	0.47257	0.4903	0.4131	0.2417	**7.07E-05**
PPARGC1A	chr4:23814301:T:C/A rs2970847	T	acA/acG p.Thr394Thr	ns	C:1.605	0.17626	0.1408	0.1923	**0.00077**	0.1791
PPARGC1A	chr4:23814039:C:T rs8192678	T	Ggt/Agt p.Gly482Ser	ns	T:16.24	0.35721	0.4272	0.3450	**3.33E-06**	0.3964
PPARGC1A	chr4:23813899:C:T/A/G rs3755863	T C in TSI	acG/acA p.Thr528Thr	ns	T:9.102	0.43796	0.5097	0.4054	**2.42E-06**	**0.02870**

Genomic coordinates from NCBI Build GRCh38.p13 (hg38) and dbSNP refSNP (rs) identifiers from build 155 are provided. mRNA levels significantly affected by SNPs in vascular and/or brain tissues (reported in GTEx portal as expression quantitative traits, eQTL) are provided. ns, no significant eQTL was found. NPHP5, a nephrocystin protein that interacts with calmodulin and the retinitis pigmentosa GTPase regulator protein; CASC3, exon junction complex subunit GJD3, involved into spliceosomes; WIPF2, WAS/WASL interacting protein family member 2, involved in the WASP-mediated organization of the actin cytoskeleton; VAMP3, a protein involved in docking/fusion of synaptic vesicles; UTS2, urotensin 2, a vasoconstrictor peptide. Estimated effect on protein function was assessed with the Combined Annotation Dependent Depletion (CADD) phred-scale scores v1.4. Minor allele frequency (MAF) from WES of affected subjects collected by the Network for Italian Genomes (NIG-IT); controls from 1000 Genome Tuscany (TSI) and gnomAD 3.1.1 non-Finnish European (nfe) databases are given. ($) In RED SNPs significant after Bonferroni correction; In BLUE SNPs at borderline significance after Bonferroni correction; in BLACK variants nominally significant. (*) Intronic localization in the partially overlapping *ILDR1*.
