# Supplementary material for: Combination of Genomic and Transcriptomic Approaches Highlights Vascular and Circadian Clock Components in Multiple Sclerosis
Source: Int J Mol Sci. 2021 Dec 28;23(1):310. doi: 10.3390/ijms23010310 (PMC8745220; doi:10.3390/ijms23010310)
Supplement: Supplementary file 1 [file ijms-23-00310-s001.zip › Table_S2.pdf]

Table S2. Predicted functional partners of proteins encoded by MS-GWAS genes differentially expressed between MS- and control IJV walls: association with neuronal/vascular traits/diseases.

Proteins (GWAs Genes)	Functional Partners *	Processes/Traits/Diseases: vascular	Processes/Traits/Diseases: neuronal
CAMK2G	GluN2A (GRIN2A)	cerebral ischaemia [1]	synaptic plasticity, epilepsy and intellectual disability, brain volume in MS [2-3]
	GluN2B (GRIN2B)	proliferation and migration of arterial VSMC, PAH, ischemic vascular dementia [4-5]	brain development, multiple neurodevelop-mental disorders, brain volume in AD [6]
IL20RA	IL20RB	serum sphingomielin levels [7] (PLOS Genet 2008)	Neuroinflammation in EAE [8]
	IL-26	nr	CNS barrier regulation [9]
LEF-1	PITX2 #	atrial fibrillation, cerebral small-vessel disease [10]	axial development of mammalian brain [11]
Rev-ErbA-α (NR1D1)	PGC-1α # (PPARGC1A)	heart development, angiogenesis, atherosclerosis [12-13]	ALS, PD, AD, decreased PPARGC1A mRNA in RR-MS, neuronal loss in MS cortex [14-15]
TEF	PER3 #	cardiac functions, development and progression of MI [16-17]	sleep disturbance in MS [18]

*When different, the name of the corresponding gene is reported in parentheses. # transcriptional regulator; GRIN2A=Glutamate receptor ionotropic, NMDA 2A (GluN2A protein); GRIN2B = glutamate ionotropic receptor NMDA 2B (GluN2B protein); PITX2 (gene) Pituitary homeobox 2 (protein)**;** PPARGC1A= Peroxisome proliferator-activated receptor gamma coactivator 1-alpha (PPARGC-1-alpha protein).; PAH, pulmonary arterial hypertension; PD =Parkinson's disease; nr= not reported in literature. NR1D1 is also a functional partner of TEF.

[1] Sun Y, Cheng X, Hu J, Gao Z. The Role of GluN2A in Cerebral Ischemia: Promoting Neuron Death and Survival in the Early Stage and Thereafter. Mol Neurobiol. 2018 Feb;55(2):1208-1216. doi: 10.1007/s12035-017-0395-8. Epub 2017 Jan 19. PMID: 28102473.

[2] Strijbis EM, Inkster B, Vounou M, Naegelin Y, Kappos L, Radue EW, Matthews PM, Uitdehaag BM, Barkhof F, Polman CH, Montana G, Geurts JJ. Glutamate gene polymorphisms predict brain volumes in multiple sclerosis. Mult Scler. 2013 Mar;19(3):281-8. doi: 10.1177/1352458512454345.

[3] Sun Y, Cheng X, Hu J, Gao Z. The Role of GluN2A in Cerebral Ischemia: Promoting Neuron Death and Survival in the Early Stage and Thereafter. Mol Neurobiol. 2018 Feb;55(2):1208-1216. doi: 10.1007/s12035-017-0395-8.

[4]. Quatredeniers M, Nakhleh MK, Dumas SJ, Courboulin A, Vinhas MC, Antigny F, Phan C, Guignabert C, Bendifallah I, Vocelle M, Fadel E, Dorfmüller P, Humbert M, Cohen-Kaminsky S. Functional interaction between PDGFβ and GluN2B-containing NMDA receptors in smooth muscle cell proliferation and migration in pulmonary arterial hypertension. Am J Physiol Lung Cell Mol Physiol. 2019 Mar 1;316(3):L445-L455

[5] Mohamed NE, Lee JH, Francis PT, Esiri MM, Chen CP, Lai MK. Differential alterations of neocortical GluN receptor subunits in patients with mixed subcortical ischemic vascular dementia and Alzheimer's disease. J Alzheimers Dis. 2015;44(2):431-7.

[6] Hu C, Chen W, Myers SJ, Yuan H, Traynelis SF. Human GRIN2B variants in neurodevelopmental disorders. J Pharmacol Sci. 2016 Oct;132(2):115-121. doi: 10.1016/j.jphs.2016.10.002. Epub 2016 Oct 19. Erratum in: J Pharmacol Sci. 2017 Apr;133(4):280. PMID: 27818011; PMCID: PMC5125235.

[7]. Gieger C, Geistlinger L, Altmaier E, Hrabé de Angelis M, Kronenberg F, Meitinger T, Mewes HW, Wichmann HE, Weinberger KM, Adamski J, Illig T, Suhre K. Genetics meets metabolomics: a genome-wide association study of metabolite profiles in human serum. PLoS Genet. 2008 Nov;4(11):e1000282. doi: 10.1371/journal.pgen.1000282

[8] Dayton JR, Yuan Y, Pacumio LP, Dorflinger BG, Yoo SC, Olson MJ, Hernández-Suárez SI, McMahon MM and Cruz-Orengo L (2021) Expression of IL-20 Receptor Subunit β Is Linked to EAE Neuropathology and CNS Neuroinflammation. Front. Cell. Neurosci. 15:683687.

[9] Broux B, Zandee S, Gowing E, Charabati M, Lécuyer MA, Tastet O, Hachehouche L, Bourbonnière L, Ouimet JP, Lemaitre F, Larouche S, Cayrol R, Bouthillier A, Moumdjian R, Lahav B, Poirier J, Duquette P, Arbour N, Peelen E, Prat A. Interleukin-26, preferentially produced by TH17 lymphocytes, regulates CNS barrier function. Neurol Neuroimmunol Neuroinflamm. 2020 Aug 11;7(6):e870.

[10] French CR, Seshadri S, Destefano AL, Fornage M, Arnold CR, Gage PJ, Skarie JM, Dobyns WB, Millen KJ, Liu T, Dietz W, Kume T, Hofker M, Emery DJ, Childs SJ, Waskiewicz AJ, Lehmann OJ. Mutation of FOXC1 and PITX2 induces

cerebral small-vessel disease. J Clin Invest. 2014 Nov;124(11):4877-81. doi: 10.1172/JCI75109. PMID: 25250569; PMCID: PMC4347243.
[11] Waite MR, Martin DM. Axial level-specific regulation of neuronal development: lessons from PITX2. J Neurosci Res. 2015 Feb;93(2):195-8. doi: 10.1002/jnr.23471. PMID: 25124216.
[12] Kadlec AO, Chabowski DS, Ait-Aissa K, Gutterman DD. Role of PGC-1α in Vascular Regulation: Implications for Atherosclerosis. Arterioscler Thromb Vasc Biol. 2016 Aug;36(8):1467-74. doi: 10.1161/ATVBAHA.116.307123. Epub 2016 Jun 16. PMID: 27312223; PMCID: PMC4965312.
[13] Chambers JM, Wingert RA. PGC-1α in Disease: Recent Renal Insights into a Versatile Metabolic Regulator. Cells. 2020 Oct 3;9(10):2234.
[14] Kolić I, Stojković L, Dinčić E, Jovanović I, Stanković A, Živković M. Expression of LEP, LEPR and PGC1A genes is altered in peripheral blood mononuclear cells of patients with relapsing-remitting multiple sclerosis. J Neuroimmunol. 2020 Jan 15;338:577090. doi: 10.1016/j.jneuroim.2019.577090.
[15] Witte ME, Nijland PG, Drexhage JA, Gerritsen W, Geerts D, van Het Hof B, Reijerkerk A, de Vries HE, van der Valk P, van Horssen J. Reduced expression of PGC-1α partly underlies mitochondrial changes and correlates with neuronal loss in multiple sclerosis cortex. Acta Neuropathol. 2013 Feb;125(2):231-43. doi: 10.1007/s00401-012-1052-y.
[16] Lipkova J, Splichal Z, Bienertova-Vasku JA, Jurajda M, Parenica J, Vasku A, Goldbergova MP. Period3 VNTR polymorphism influences the time-of-day pain onset of acute myocardial infarction with ST elevation. Chronobiol Int. 2014 Oct;31(8):878-90. doi: 10.3109/07420528.2014.921790. PMID: 24866331.[
[17] Kovac U, Jasper EA, Smith CJ, Baer RJ, Bedell B, Donovan BM, Weathers N, Prosenc Zmrzljak U, Jelliffe-Pawlowski LL, Rozman D, Ryckman KK. The Association of Polymorphisms in Circadian Clock and Lipid Metabolism Genes With 2nd Trimester Lipid Levels and Preterm Birth. Front Genet. 2019 Jun 13;10:540. doi: 10.3389/fgene.2019.00540. PMID: 31249592; PMCID: PMC6584752
[18] Golalipour M, Maleki Z, Farazmandfar T, Shahbazi M. *PER3* VNTR polymorphism in Multiple Sclerosis: A new insight to impact of sleep disturbances in MS. Mult Scler Relat Disord. 2017 Oct;17:84-86. doi: 10.1016/j.msard.2017.07.005.
